# A Thermoregulatory Flexible Phase Change Nonwoven for All-Season High-Efficiency Wearable Thermal Management

**DOI:** 10.1007/s40820-022-00991-6

**Published:** 2023-01-04

**Authors:** Hanqing Liu, Feng Zhou, Xiaoyu Shi, Keyan Sun, Yan Kou, Pratteek Das, Yangeng Li, Xinyu Zhang, Srikanth Mateti, Ying Chen, Zhong-Shuai Wu, Quan Shi

**Affiliations:** 1 Liaoning Province Key Laboratory of Thermochemistry for Energy and Materials, Dalian National Laboratory for Clean Energy, Dalian Institute of Chemical Physics, Chinese Academy of Sciences, 457 Zhongshan Road, Dalian, 116023 People’s Republic of China; 2 State Key Laboratory of Catalysis, Dalian Institute of Chemical Physics, Chinese Academy of Sciences, 457 Zhongshan Road, Dalian, 116023 People’s Republic of China; 3 Institute for Frontier Materials, Deakin University, Waurn Ponds, VIC 3216 Australia; 4 University of Chinese Academy of Sciences, 19 A Yuquan Road, Shijingshan District, Beijing, 100049 People’s Republic of China

**Keywords:** Phase change materials, Graphene, Boron nitride, Nonwoven, Wearable thermal management

## Abstract

Phase change materials have a key role for wearable thermal management, but suffer from poor water vapor permeability, low enthalpy value and weak shape stability caused by liquid phase leakage and intrinsic rigidity of solid–liquid phase change materials. Herein, we report for the first time a versatile strategy for designed assembly of high-enthalpy flexible phase change nonwovens (GB-PCN) by wet-spinning hybrid graphene-boron nitride (GB) fiber and subsequent impregnating paraffins (e.g., eicosane, octadecane). As a result, our GB-PCN exhibited an unprecedented enthalpy value of 206.0 J g^−1^, excellent thermal reliability and anti-leakage capacity, superb thermal cycling ability of 97.6% after 1000 cycles, and ultrahigh water vapor permeability (close to the cotton), outperforming the reported PCM films and fibers to date. Notably, the wearable thermal management systems based on GB-PCN for both clothing and face mask were demonstrated, which can maintain the human body at a comfortable temperature range for a significantly long time. Therefore, our results demonstrate huge potential of GB-PCN for human-wearable passive thermal management in real scenarios.

**Supplementary Information:**

The online version contains supplementary material available at 10.1007/s40820-022-00991-6.

## Highlights

- The first assembled flexible phase change nonwoven is reported by wet-spinning.
- The unprecedented thermal properties of such flexible phase change nonwoven are achieved.
- Such phase change nonwoven is highly applicable for mask and cloth intelligent temperature control.

**Supplementary Information:**

The online version contains supplementary material available at 10.1007/s40820-022-00991-6.

## Graphical abstract

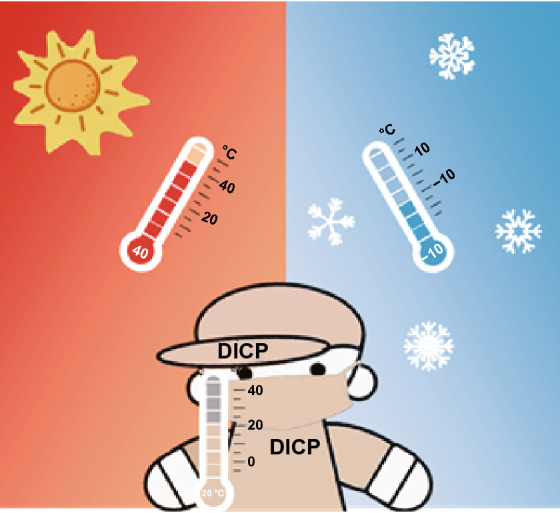

## Introduction

Rapidly growing demand for wearable thermal energy management systems in various applications [1, 2], such as wearable sensors [3, 4], supercapacitors [5, 6] and clothing [7, 8], has accelerated the development of flexible multifunctional phase change materials (PCMs) [9]. In particular, organic PCMs (such as polyethylene glycol (PEG) [10], paraffin wax (PW) [11, 12] and fatty amine [13]) based on temperature-regulating composites have garnered widespread attention, owing to their high heat storage density [14], excellent chemical and thermal stability [15], nontoxicity [16], environmental friendliness [17], approximately constant-temperature phase change process [18] in human wearable thermal management [19]. However, these conventional phase change composites suffer from inherent shortcomings, [20], such as liquid leakage [21], strong mechanical rigidity [22] and poor water vapor permeability [23], substantially preventing their practical application in wearable thermal energy management [24]. To this end, two key strategies have been developed for temperature-regulating composites so far [25]. One is a method recently reported by our group [26] for synthesizing intrinsically flexible PEG films by chemical cross-linking, demonstrating adjustable phase change temperature and enthalpy, excellent flexibility and shape stability. However, this flexible phase change film displayed poor water vapor permeability and low thermal energy storage density. Another strategy is to fabricate flexible phase change fibers [27], mainly prepared by directly encapsulating PCMs into fibers through electrospinning technology [28]. As an example, Wan et al*.* [29] prepared polyacrylonitrile/isopropyl palmitate sheath/core-shaped nanofibers with various phase change temperature. Similarly, Lu et al*.* [30] developed core-sheath structured composite films with PW as core and polyacrylonitrile as sheath. However, these reported flexible phase change fabrics via one-step electrospinning process also exhibit low enthalpy value of less than 150 J g^−1^ [31]. Therefore, to further meet practical requirement, the designed fabrication of high-enthalpy phase change fabric with wearability, flexibility, outstanding water vapor permeability and anti-leakage capability is urgently needed.

Hence, we report, for the first time, the designed assembly of graphene–boron nitride (GB) phase change nonwoven (GB-PCN) by wet-spinning and vacuum impregnation for high-efficiency wearable thermal management. Firstly, a facile, mass-producible and low-cost wet-spinning strategy is proposed to construct hierarchically connected three-dimensional (3D) free-standing flexible GB nonwovens. Subsequently, the PCMs (e.g., eicosane, octadecane) were impregnated into the GB nonwovens with abundant mesopores to prepare the shape-stable GB-PCN. The optimized GB-PCN delivered an ultrahigh enthalpy and high loading of 206 J g^−1^ and 83%, respectively. Further, GB-PCN displays an excellent thermal conductivity and solar-thermal conversion ability, superior water vapor permeability (close to cotton) and hydrophobicity (136°). In addition, GB-PCN showed excellent temperature control performance in the human-wearable intelligent temperature-regulating clothes for about 24 min. And the smart thermal management face mask prepared using GB-PCNs can keep warm in winter and cool in summer for about 19 min.

## Experimental Section

### Materials

Eicosane (AR) and octadecane (AR) were obtained from Aladdin Reagent (Shanghai, China). Stannous chloride (SnCl_2_·2H_2_O) (AR) was purchased from Guoyao Chemical Co., Ltd. All the reagents were used without any further treatment.

### Synthesis of the GO-BN Hybrid Nonwoven

Graphene oxide (GO) suspension was prepared by a modified Hummers method [29], and boron nitride (BN) nanosheets were was prepared by ball-milling-assisted method [30]. Subsequently, a uniform hybrid suspension containing GO (10 mg mL^−1^) and BN nanosheets with the mass radio of 2:1, 4:1 and 1:0 was prepared by sonication for 60 min and stirring for 30 min. Next, the mixed suspension was spun into 1-ethyl-3-methylimidazolium (EMCl)/ethanol (1.5 g/100 mL) at a rate of 60 µL min^−1^ to form *x*GO-BN (*x*GOB) hybrid fiber, which was cut into small pieces with a scissor. Furthermore, the *x*GOB hybrid nonwoven (*x*GOB-N) fabrics could be prepared by suction filtration with short fibers. Then, the *x*GOB hybrid nonwoven was immersed in 0.1 g mL^−1^ SnCl_2_ hydrochloric acid solution (0.1 mol L^−1^) at 80 °C for 12 h. Last but not least, the xGB hybrid nonwoven was washed by deionized water for three times and freeze-dried for 24 h under vacuum.

### Preparation of Phase Change Nonwoven

The PCN was prepared using vacuum-assisted impregnation methods. Typically, both xGB nonwoven and PCMs (eicosane or octadecane) placed in one container were heated to 80 °C in a vacuum oven and maintained for 24 h for effective infusion of PCMs into the xGB hybrid nonwoven. After that, the PCN was taken out and placed on filter paper under 80 °C until the excess PCMs adhered to the PCN surface were completely removed. After cooling down to room temperature, the PCN was obtained.

### Solar-Thermal Energy Conversion Measurement

Solar–thermal energy conversion measurement was taken using the simulated sunlight provided by the xenon lamp source (Beijing Bofeilai Technology Co., Ltd., China), where the distance between the sample and the light source is set to 20 cm, and the illumination intensity is 100, 150, 200, and 300 mW cm^−2^.

### Water Vapor Transmission Rate Test

The testing procedure is based on ASTM E96 with glass bottle were filled with distilled water. The bottles were sealed by polyethylene (PE), cotton and cotton-GB-PCN, respectively. The sealed bottles were then placed into an environmental chamber in which temperature and relative humidity were held at 25 °C and at 50 ± 5%. The mass of the bottle and the sample were measured periodically, and the reduced mass was thought as the mass of the evaporated water. The reduced mass was then divided by the area to evaluate the water vapor transmission.

### Face Mask Thermal Management Experiment

Cold environment: After heating above the phase transition temperature, the author wears the PCN on the face. In the outdoor environment (3–9 °C, November 30, 2021), use an infrared camera to take infrared pictures at different times to monitor temperature changes. Hot environment: Due to the low outside temperature, we used high-flow, heated (38 °C) nitrogen gas to flow through the mask, while using an infrared camera to monitor temperature changes.

### Material Characterization

The morphology of the samples was visually characterized via scanning electron microscopy (SEM, JSM-7900) at an accelerating voltage of 20 kV. Transmission electron microscopy (TEM) measurement was taken on a Tecnai G2F20 S-Twin with an acceleration voltage of 200 kV. The pore size distribution and average pore diameter of the nonwoven were analyzed by the BJH nitrogen adsorption and desorption instrument (ASAP 2020, Micromeritics, USA). The surface area of the aerogels was determined by the Brunauer–Emmett–Teller (BET) method, based on the amount of N_2_ adsorbed at pressures 0.05 < *P*/*P*_0_ < 1. The crystal structures of the PCM composites were investigated using an automated X-ray powder diffractometer (XRD, SmartLab, Japan). The XRD patterns were tested at a scan rate of 20° min^−1^ with a 2*θ* range of 5°–80°. Raman spectra were obtained using a Renishaw Invia Raman microscope system (Renishaw, Britain) with 575 nm laser excitation. The chemical compositions of the PCM composites were investigated using Fourier transform infrared (FTIR) spectroscopy. The phase change properties of the composite PCMs were estimated using a differential scanning calorimeter (DSC, Discovery DSC from TA Instruments), and the test was carried out from − 10 to 80 °C with a heating and cooling rate of 10 °C min^−1^ under a nitrogen atmosphere. The mass of the samples was in the range of 5–10 mg. The measurement uncertainties of DSC measurement are within ± 0.025 °C for the temperature and within ± 0.04% for the enthalpy. Thermal gravimetric analysis (TGA) and DTG were carried out using a TG 209F1 Libra (NETZSCH) analyzer with a heating rate of 10 °C min^−1^ in a nitrogen atmosphere. The masses of the samples were about 10–20 mg.

## Results and Discussion

### Preparation of the GB-PCN

**Fig. 1:**
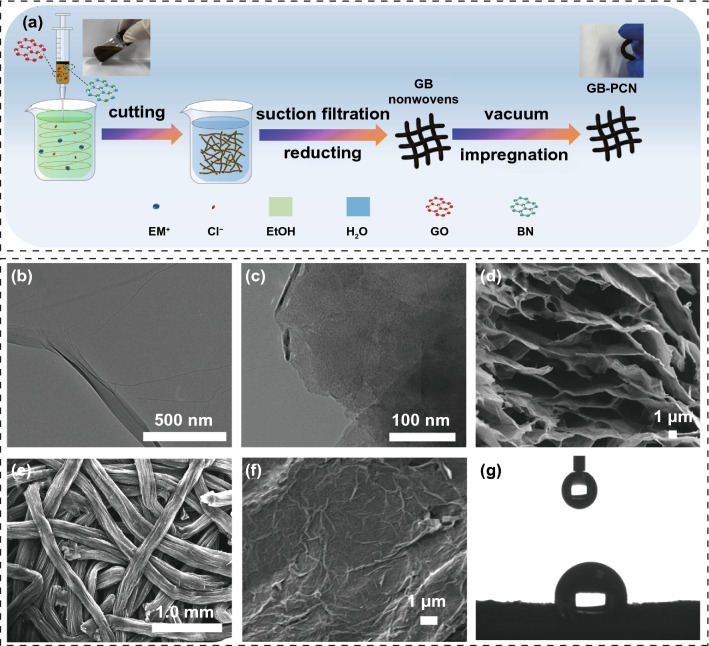
Schematic diagram of the preparation process and structural characterizations for GB-PCN. **a** Schematic illustrating the fabrication of GB-PCN nonwoven. Inset images are the photographs of GO-BN spinning solution (left) and GB-PCN (right). TEM images of **b** GO nanosheets and **c** BN nanosheets. **d** Cross-sectional SEM image of a GB fiber. **e**, **f** Top-view SEM images of GB-PCN. **g** Photograph of the contact angle measurement of GB-PCN

The fabrication process for hybrid GB-PCN is schematically in Fig. 1a. First, GO and BN nanosheets are synthesized [32–34]. It can be seen from TEM images (Fig. 1b, c) that both of GO and BN nanosheets have a few-layer structure, proving a high degree exfoliation. After homogeneously mixing of these two 2D nanosheets solutions (Fig. 1a, inset image), GB-N was prepared by wet spinning, filtration, and reduction process. The cross-sectional SEM image (Fig. 1d) of GB fiber shows that the nanosheets were stacked and wrapped in parallel, aligned with the fiber’s long axis owing to the shear force during wet spinning, which is highly conducive to the transmission and utilization of thermal energy. Further, the GB-PCN fabric was obtained by vacuum-assisted impregnation of the GB-N and alkane under heating treatment at 80 °C. Figure 1e shows that the GB-PCN has an interlinked network between fibers, which enhances the mechanical strength of the overall structure and establishes a wide thermal energy transmission network. High-magnification SEM image (Fig. 1f) shows that the surface of the GB-PCN fabric is flat. Furthermore, it is worth mentioning that the GB-PCN displays satisfactory flexibility (Fig. 1a), an important parameter in practical applications. In addition, hydrophobicity is also vital for fabrics, which could endow the cloth with outstanding self-cleaning [35]. In order to study the hydrophilic and hydrophobic properties of the GB-PCN, static water contact angle measurements were taken at room temperature. As shown in Fig. 1g, the contact angle of water droplets on GB-PCN is 136° (more than 90°), confirming the hydrophobic nature of the GB-PCN.

### Structural Characterizations and Thermal Properties of GB-PCN

**Fig. 2:**
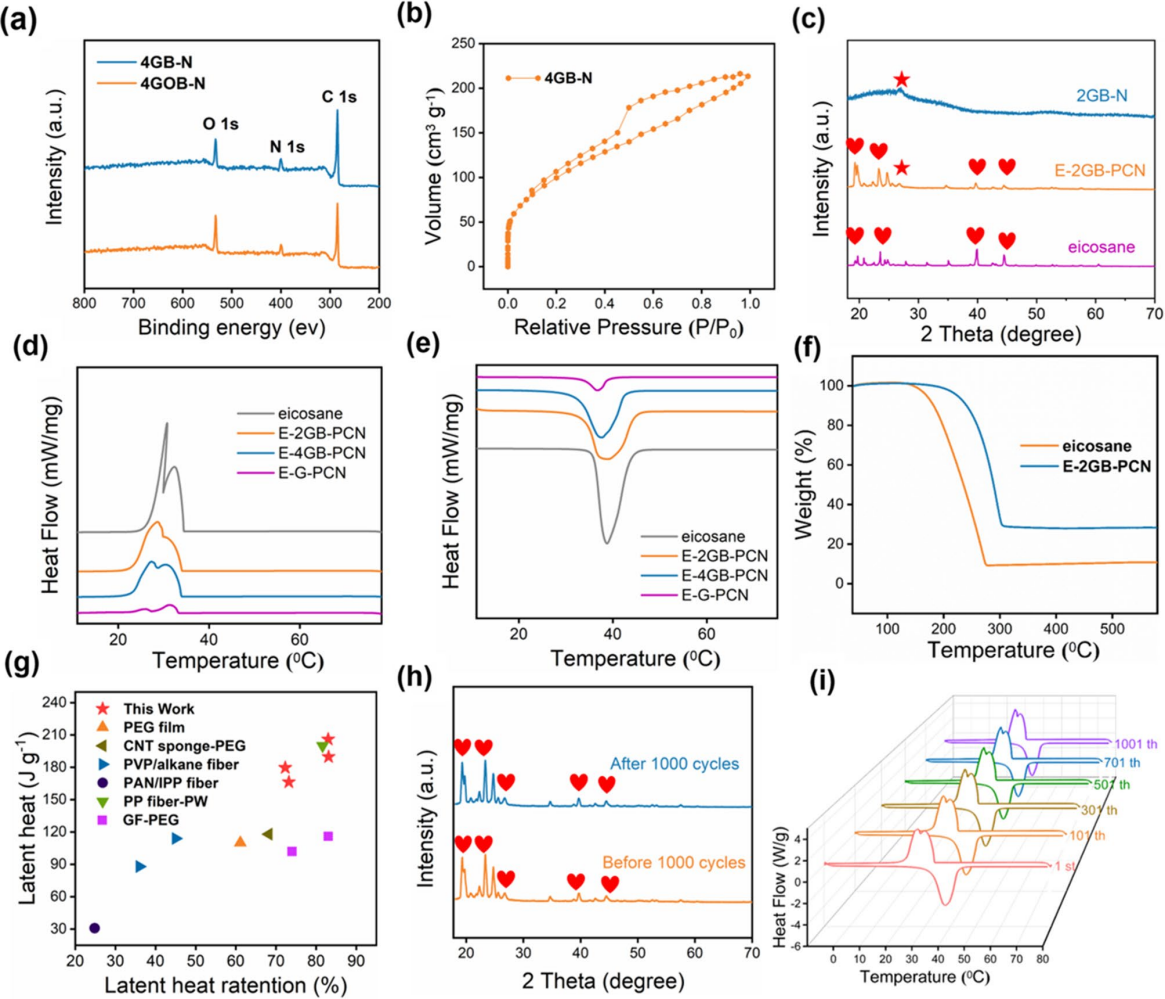
Structural characterizations and thermal properties of GB-PCN. **a** XPS spectra of GOB-N and GB-N before and after reduction. **b** Nitrogen adsorption and desorption isotherm of 4 GB-N. **c** XRD patterns of eicosane, 2 GB-N and E-2 GB-PCN. **d**, **e** DSC curves of eicosane, E–G-PCN, E-4 GB-PCN and E-2 GB-PCN. (f) TG curves of eicosane and E-2 GB-PCN. **g** Comparisons of the enthalpy value and the latent heat retention of GB-PCN with the reported PCMs. **h** XRD patterns of E-4 GB-PCN before and after 1000 cycles. **i** Cycling stability of E-4 GB-PCN

Raman spectroscopy was applied to explore the structure evolution from GOB-N to GB-N (Fig. S1), in which two significant peaks are observed at approximately 1580 cm^−1^ (G peak) and 1340 cm^−1^ (D peak). Meanwhile, GOB-N shows a sharp D peak while the corresponding peak for the GB-N was weak after chemical reduction. Furthermore, X-ray photoelectron spectroscopy (XPS) (Figs. 2a and S2) confirms the increased C/O ratio of 4.63 for GOB-N to 7.28 for GB-N due to the significant removal of oxygen-functional groups, suggestive of efficient reduction of GOB-N into GB-N.

The nitrogen adsorption and desorption isotherm of the GB-N show a Type-*IV* curve with an H3 hysteresis loop (Fig. 2b). It can be seen that the capillary agglomeration occurs at a relative pressure of 0.5 < *P/P*_0_ < 1.0, and the amount of adsorption increases sharply, indicative of the presence of mesopores/macropores. According to the amount of gas adsorbed, the pore volume and specific surface area of GB-N were calculated to be 0.324 cm^3^ g^−1^ and 325 m^2^ g^−1^, respectively. In addition, the pore size distribution of the GB-N gives an average pore size around 4.1 nm (Fig. S3), characteristic of mesopores, which is the key to confine the melted PCMs. Furthermore, XRD patterns (Fig. 2c) validated the phase structures of 2 GB-N, eicosane and eicosane-based 2 GB-PCN (E-2 GB-PCN). Apparently, no new peaks appeared in the XRD pattern of E-2 GB-PCN except those of 2 GB-N and eicosane, indicative of physical interaction connection between 2 GB-N and eicosane. Moreover, to further explore the interaction between the carrier material and PCM, we also tested the FT-IR spectra (Fig. S10) of eicosane, 2 GB-N and E-2 GB-PCN. It is confirmed that there is no new chemical bond formed in the E-2 GB-PCN composite, indicating that the combination of PCM and 2 GB-N carrier is likely from physical interaction.

For solid–liquid PCMs, the liquid leakage is a huge obstacle hindering practical application. Compared with pure eicosane, a good anti-leakage capability of E-GB-PCNs upon heating at 80 °C is confirmed by the leakage test (Table S1). In the initial state, both samples are solid state. After heating for a few minutes, the eicosane began to melt, and after heating for 10 min, the pure eicosane was completely melted and overspread on the filter paper. On the other hand, GB-PCN could retain its initial shape owing to the confinement of the liquefied eicosane in the mesopores of GB-N. Furthermore, in comparison with pure eicosane, the quality of the filter paper of eicosane-based 2 GB (mass ratio of G:B is 2:1)-PCN (E-2 GB-PCN) and eicosane-based 4 GB (mass ratio of G:B is 4:1)-PCN (E-4 GB-PCN) before and after melting had no significant change, further confirming excellent anti-leakage capability of GB-PCN.

Latent heat is the most fundamental parameter to assess the performance of phase change composites, which is closely related to the energy density of thermal management systems. Owing to the presence of abundant mesopores inside GB-N, we realized an ultrahigh enthalpy value of 206 J g^−1^ (Fig. 2d, e), far beyond the normal phase change fibers (< 150 J g^−1^) [27, 28]. The detailed phase change parameters were extracted from the DSC curves, such as melting/cooling temperature (*T*_m_/*T*_c_) and enthalpy (Δ*H*_m_/Δ*H*_c_), which are presented in Table S3. To the best of our knowledge, our GB-PCN exhibiting a superb enthalpy value of 206 J g^−1^ and superior latent heat retention of 83% (Fig. 2g) far exceed the performance of previously reported phase change fibers or phase change films (Table S5), such as flexible melamine-toluene diisocynate-PEG (MTPEG) phase change films (110 J g^−1^) [26], CNT sponge-PEG film(118 J g^−1^) [19], commercial hollow polypropylene (PP) fiber-PW (199.9 J g^−1^) [36], graphene fiber (GF)—PEG (124 J g^−1^) [7], polyvinyl pyrrolidone (PVP)/alkane fiber (114 and 88 J g^−1^) [31], and polyacrylonitrile (PAN)/isopropyl palmitate (IPP) fiber (30.8 J g^−1^) [29].

A better thermal stability of GB-PCN compared to pure PCMs is verified through thermogravimetric analysis. According to thermogravimetric curves in Fig. 2f**,** the E-2 GB-PCN experiences a major weight loss between 200 and 300 °C, while the weight loss of pure eicosane mainly occurs between 150 and 250 °C. The superior thermal stability of 2 GB-PCN over that of pure PCMs is likely due to the capillary force between the eicosane and GB-N [37]. The long-term usability of GB-PCN as fabrics for clothing depends largely on the thermal cycling capacity of phase change composites. Cycling tests were conducted to judge the thermal durability of GB-PCN and evaluated based on the changes of phase transition temperature and enthalpy value at different thermal cycles. From the DSC curves in Fig. 2i, it can be seen that after 1000 thermal cycles, the melting and crystallization temperature and latent heat had little change, manifesting excellent thermal cycle stability of GB-PCN. In addition, there is no prominent change in the shape and position of the diffraction peaks through XRD patterns for E-4 GB-PCN before and after 1000 thermal cycles. Moreover, to prove wide applicability of GB nonwoven for supporting other PCMs for different demands, we further adopt a similar strategy to fabricate the GB-PCN using octadecane as thermal energy storage material (Figs. S4, S6, S7, S8 and Table 2). The composites present the phase change temperature of 22.7 °C and high enthalpy value of 188.7 J g^−1^.

### Solar–Thermal Conversion of GB-PCN

**Fig. 3:**
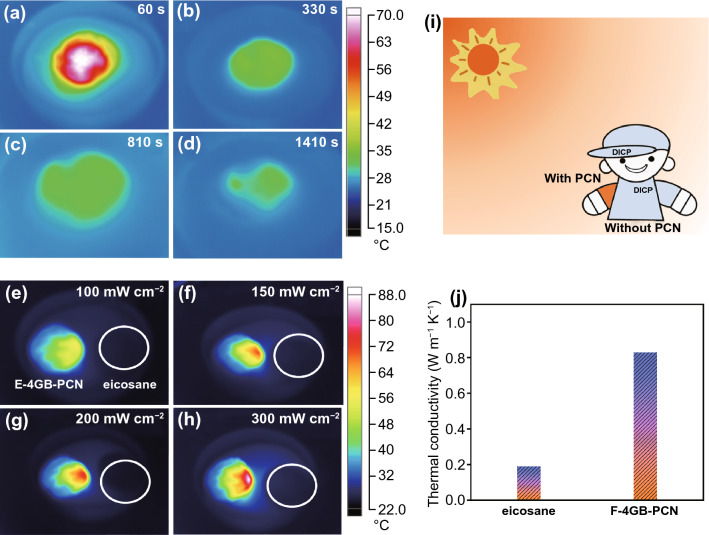
Solar–thermal conversion of GB-PCN. **a**–**d** Infrared imaging pictures of the solar–thermal conversion process of E-4 GB-PCN under photo-illumination of 200 mW cm^−2^ for different times. **e**–**h** Infrared imaging pictures of the solar–thermal conversion process of E-4 GB-PCN and eicosane under different illumination intensity for 90 s. **i** Schematic diagram of solar–thermal conversion in the cloth (GB-PCN in the clothing on the left arm, and no GB-PCN in the clothing on the right arm). **j** Thermal conductivity of eicosane and E-4 GB-PCN

To evaluate the solar–thermal conversion performance, the light absorption capacity of eicosane and composite PCM was measured by UV–Vis spectrophotometry. As shown in Fig. S9, it is clearly seen that the light absorption capacity of the E-2 GB-PCN is significantly better than that of eicosane in the wavelength range of 400–800 nm. It is indicated that the graphene-BN nonwoven support has an ultra-high light absorption capability, which enables the PCM composite to capture light energy more efficiently and further convert it into thermal energy owing to the efficient solar–thermal conversion. To verify the solar–thermal energy conversion, storage and release ability of the GB-PCN, we conducted corresponding solar–thermal conversion measurement. The GB-PCN was placed under the simulated solar illumination with intensity of 200 mW cm^−2^ (2 sun), and the light remained turned on for 60 s. Impressively, the temperature of GB-PCN still remains at 31.4 °C after 1410 s (Fig. 3a–d). Furthermore, the temperature of GB-PCN rises rapidly under solar radiation compared to pure PCM, which is attributed to the function of graphene nanosheets as effective photon traps. It is also evidenced that GB-PCN displays excellent light absorption and heat storage capabilities. After irradiation for 90 s, the temperature of GB-PCN gradually increases significantly with the increase of irradiation intensity (Fig. 3e–h). When the solar intensity is adjusted from 1 to 3 sun, the temperature rise rate becomes higher (from 50 to 80 °C), indicating that high-intensity light is conducive to faster solar–thermal conversion and storage. In short, GB-PCN is very promising for spontaneous and efficient absorption of the energy of sunlight to store thermal energy (Fig. 3i).

To utilize thermal energy more efficiently, higher thermal conductivity (*κ*) is a fundamental condition. The κ of pure E and GB-PCN was evaluated (Fig. 3j), which was significantly increased to 0.83 W m^−1^ K^−1^ for E-4 GB-PCN. This is mainly owing to the addition of high thermal conductivity BN nanosheets and the rational construction of 3D thermal pathways in GB  nonwoven.

### Wearable Thermal Management in Clothing

**Fig. 4:**
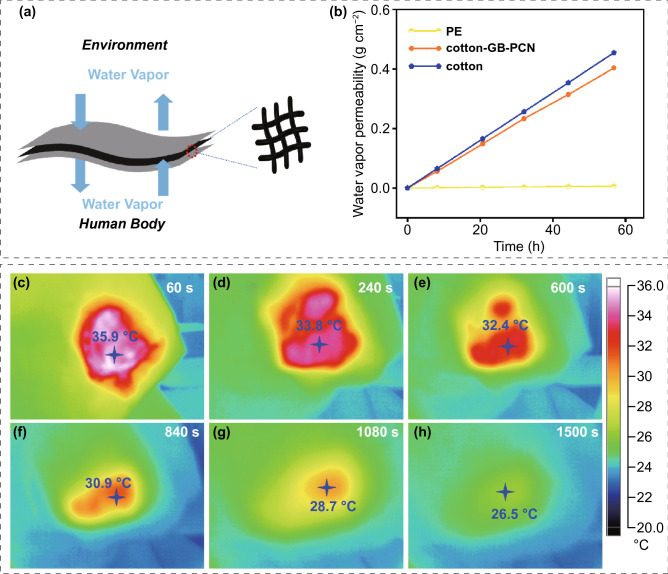
Wearable thermal management in clothing. **a** Schematic of sandwich-type human thermal management devices. **b** Water vapor transmission of polyethylene (PE), cotton and cotton-E-2 GB-PCN. IR images of E-2 GB-PCN used for clothing thermal management at different time of **c** 60 s, **d** 240 s, **e** 600 s, **f** 840 s, **g** 1080 s, and **h** 1500 s

Due to the outstanding anti-leakage capability, excellent phase change property, long cycle performance and good thermal stability, the GB-PCN with phase change temperatures of 27.3–31.3 °C holds great potential to construct wearable thermal management cloths with the appropriate working temperature of 20–36 °C [38, 39]. To this aim, we fabricated a sandwich-like thermal energy storage and temperature control device through the layer-by-layer assembly of cotton/GB-PCN/cotton film (3 × 9 cm^2^), in which graphene was selected as the light absorption unit and BN as the thermally conductive filler, eicosane as the heat storage unit, and cotton as package cloth to further increase the comfort of the human body (Fig. 4a). First, under the light intensity of 200 mW cm^−2^, the clothes are heated to above 40 °C, and GB-PCN could release heat for about 1500 s smoothly in a narrow temperature range (Fig. 4c–h). Furthermore, water vapor transmission rate is an important indicator of the materials used in human wearable fabrics. When the human body is sweating, efficient water transmission ability can enhance the comfort degree of human body. Based on this, we quantitatively examined the water vapor permeability of polyethylene (PE), cotton and cotton-GB-PCN-cotton [40]. After measuring the loss of quality of the bottle every few hours, the relationship between mass and time is almost linear (Fig. 4b). It is revealed that the water vapor transmission rate of the cotton-GB-PCN-cotton reaches 0.00709 g cm^−2^ h^−1^, which is very close to 0.00799 g cm^−2^ h^−1^ of cotton. The results show that the PE material basically has no water vapor permeability, while cotton-GB-PCN has a good water vapor permeability. Therefore, it is demonstrated that under the condition of natural convection, the GB-PCN has outstanding water vapor permeability.

### Wearable Thermal Management in Face Masks

**Fig. 5:**
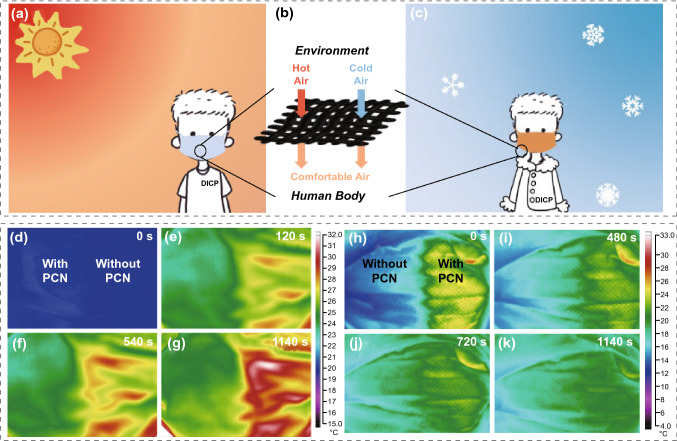
Wearable thermal management in face masks. **a**–**c** Schematic of GB-PCN used for face mask thermal management. The simulative effect through *IR* images of O-2 GB-PCN for face mask thermal management at different times of **d** 0 s, **e** 120 s, **f** 540 s, **g** 1140 s under ambient temperature of 38 °C (left part is the functionalized face mask with O-2 GB-PCN, right part is a traditional face mask without O-2 GB-PCN). The actual effect through *IR* images of E-2 GB-PCN for face mask thermal management at different time of **h** 0 s, **i** 480 s, **j** 720 s, **k** 1140 s under ambient temperature about 4 °C (right part is the functionalized face mask with E-2 GB-PCN, left part is a traditional face mask without E-2 GB-PCN)

Owing to excellent water vapor permeability, GB-PCN fabric is demonstrated to be used in face masks to achieve the functions of keeping cool in summer and warm in winter, as shown in Fig. 5a–c. We added the E-2 GB-PCN on one side of the face mask, and the other side as a contrast. In a cold environment, there is a significant temperature difference on the two sides of the mask, corresponding to the phase change process (Fig. 5h–k). Notably, the lag time is as long as 19 min, which is consistent with previously reported level [16]. Such superior heat storage and heat preservation performance are attributed to the hierarchical structure inside the GB nonwoven fabric, and in the hybrid structure, micropores and mesopores provide numerous adsorption sites. In addition, the capillary action induced by graded pores can ensure the stability of eicosane molecules. At the same time, the interconnected graphene and BN nanosheets network could form high 3D thermal conductivity channel so that PCN could maintain temperature uniformity as much as possible. However, the traditional face mask without PCMs shows a rapid cooling process. It is validated from the experimental results that the eicosane-based face mask can provide enough thermal energy and maintain a long temperature platform to meet the basic requirements of actual use. As demonstrated, this kind of face masks in summer also can efficiently cool the incoming air and improve the comfort of human body. When the face mask is used for 19 min in an environment of 38 °C, the temperature of the face mask is still maintained at a very comfortable temperature of 25 °C (Fig. 5d–g).

## Conclusions

In summary, we report a general strategy to fabricate a novel class of GB-PCN by wet-spinning and subsequent paraffin impregnation for high-efficiency wearable thermal management system. The GB-PCN showed a surprising ultrahigh enthalpy value of 206 J g^−1^, excellent thermal reliability and anti-leakage capability, outstanding thermal cycling capability of 97.6% after 1000 cycles, and ultra-high water vapor permeability (close to cotton), outperforming PCM films and fibers reported so far. Unlike active heating or cooling devices, such as air conditioners and electric heating blankets, the GB-PCN has complete self-absorbing and self-exothermic properties in response to changes with external temperature. This spontaneous heat absorption and exothermic property could greatly save extra energy usage. Based on it, assembled GB-PCN-based wearable thermal management system for clothing and face masks can keep the human body at a comfortable temperature range. This novel strategy further expands the advanced functional application of PCMs, and it is foreseeable that GB-PCNs will have a wide range of applications in the field of human wearable passive thermal management in real-world environments.

### Supplementary Information

Below is the link to the electronic supplementary material.

Supplementary file1 (PDF 898 KB)
