# Supplementary material for: A Thermoregulatory Flexible Phase Change Nonwoven for All-Season High-Efficiency Wearable Thermal Management
Source: Nanomicro Lett. 2023 Jan 4;15:29. doi: 10.1007/s40820-022-00991-6 (PMC9813330; doi:10.1007/s40820-022-00991-6)
Supplement: Supplementary file 1 — Supplementary file1 (PDF 898 KB) [file 40820_2022_991_MOESM1_ESM.pdf]

Supporting Information for

A Thermoregulatory Flexible Phase Change Nonwoven for All-Season High-Efficiency Wearable Thermal Management

Hanqing Liu[1, 2, 4, #], Feng Zhou[2, #], Xiaoyu Shi[2], Keyan Sun[1], Yan Kou[1], Pratteek Das[2, 4], Yangeng Li[1, 4], Xinyu Zhang[1, 4], Srikanth Mateti[3], Ying (Ian) Chen[3, *], Zhong-Shuai Wu[2, *], Quan Shi[1, *]

[1] Liaoning Province Key Laboratory of Thermochemistry for Energy and Materials, Dalian National Laboratory for Clean Energy, Dalian Institute of Chemical Physics, Chinese Academy of Sciences, 457 Zhongshan Road, Dalian 116023, P. R. China

[2] State Key Laboratory of Catalysis, Dalian Institute of Chemical Physics, Chinese Academy of Sciences, 457 Zhongshan Road, Dalian 116023, P. R. China

[3] Institute for Frontier Materials, Deakin University, Waurn Ponds, Vic 3216, Australia

[4] University of Chinese Academy of Sciences, 19 A Yuquan Road, Shijingshan District, Beijing 100049, P. R. China

[#] Hanqing Liu and Feng Zhou contributed equally to this work.

* Corresponding autors. E-mail: shiquan@dicp.ac.cn (Quan Shi); wuzs@dicp.ac.cn (Zhong-Shuai Wu); ian.chen@deakin.edu.au (Ying (Ian) Chen)

Supplementary Figures and Tables

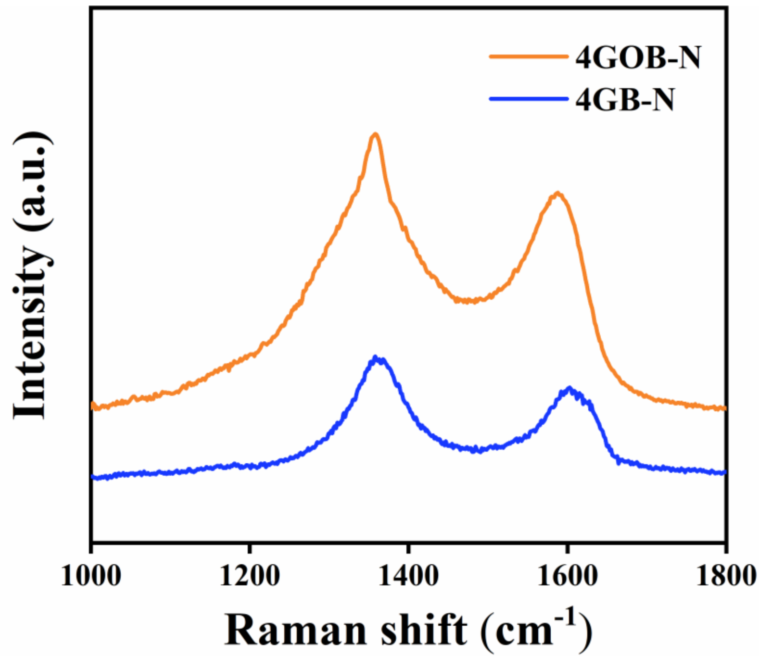

Fig. S1 Raman spectra of GB nonwoven before and after reduction

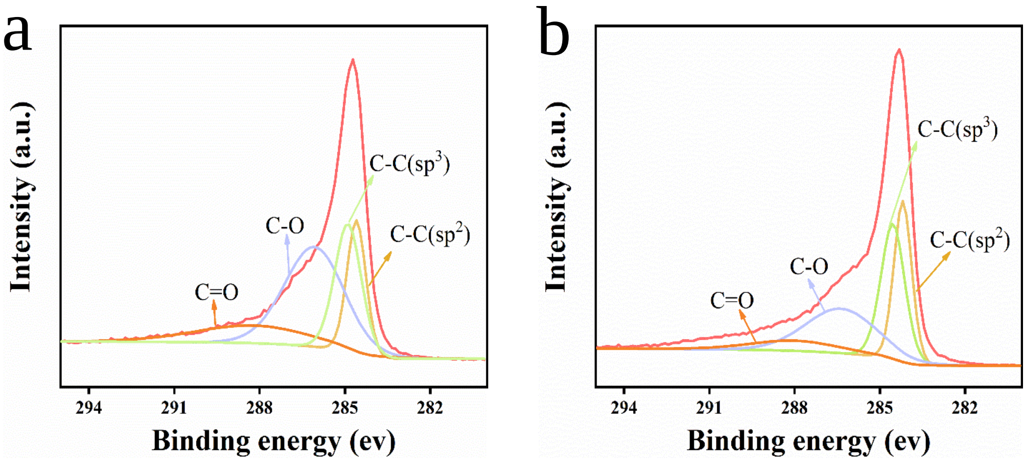

Fig. S2 C 1s XPS spectra of (**a**) GOB-N and (**b**) GB-N

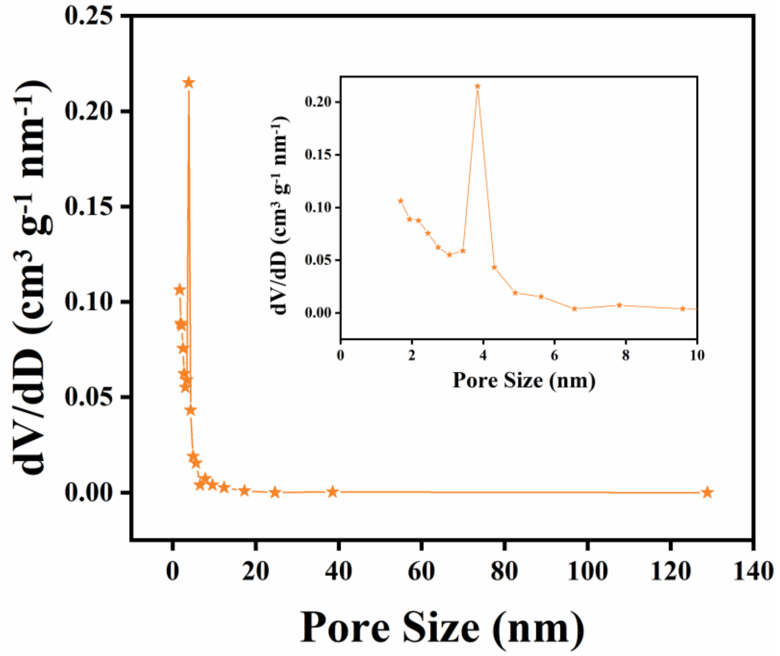

Fig. S3 Pore size distribution of 4GB-N. Inset is the partial enlarged curve from the pore size of less than 10 nm

Table S1 The mass difference before and after the leakage test of pure eicosane, E-4GB-PCN and E-2GB-PCN

**Sample**	m_{before}/g	m_{after}/g
blank filter paper	0.8015	0.8024
eicosane	0.7967	1.9445
E-4GB-PCN	0.8012	0.7990
E-2GB-PCN	0.7936	0.7973

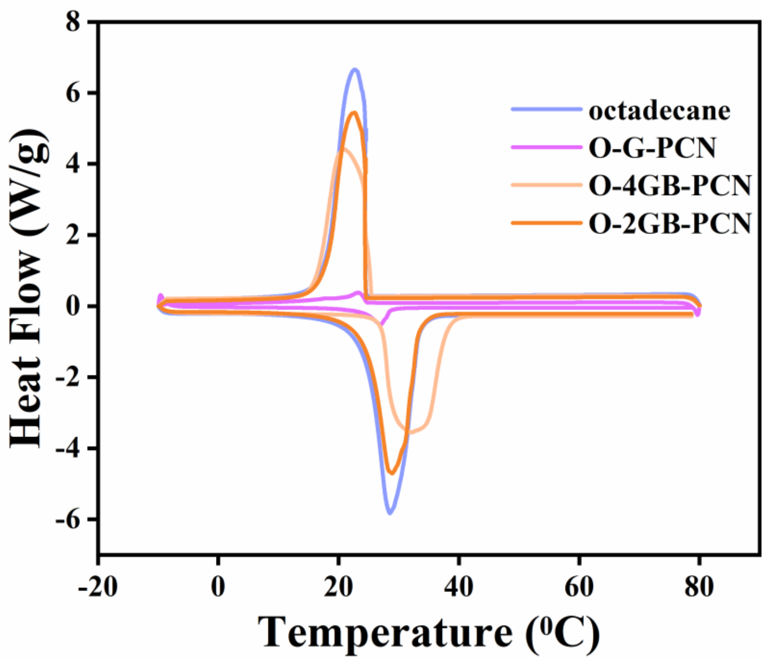

Fig. S4 DSC curves of octadecane based GB-PCN

Table S2 Thermal properties of octadecane, O-G-PCN, O-4GB-PCN and O-2GB-PCN

	T_m (°C)	$\triangle H_m$ (J/g)	T_c(°C)	$\triangle H_c$ (J/g)
octadecane	28.5	228.1	22.7	227.7
O-2GB-PCN	28.9	189.7	22.7	188.7
O-4GB-PCN	32.2	166.3	21.0	163.2
O-G-PCN	26.9	16.8	23.2	13.6

Table S3 Thermal properties of eicosane, E-G-PCN, E-4GB-PCN and E-2GB-PCN

	T_c (°C)	$\triangle H_c$ (J/g)	T_m (°C)	$\triangle H_m$ (J/g)
eicosane	30.8	245.5	38.7	248.3
E-2GB-PCN	28.6	202.4	38.8	206.0
E-4GB-PCN	27.3	175.4	37.7	179.6
E-G-PCN	31.3	30.9	36.6	32.2

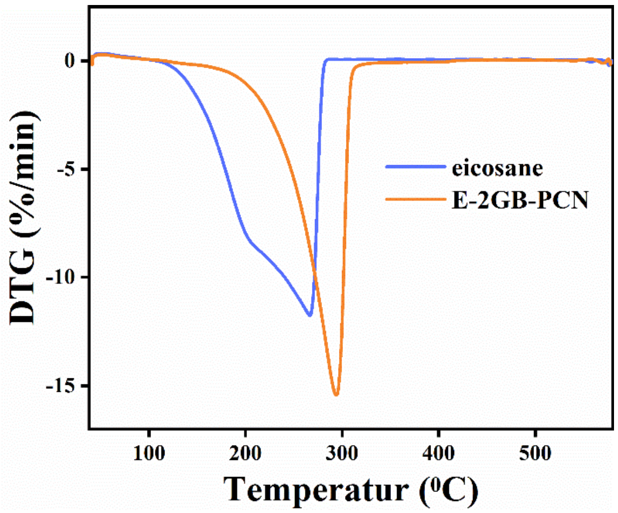

Fig. S5 DTG curves of eicosane and E-2GB-PCN

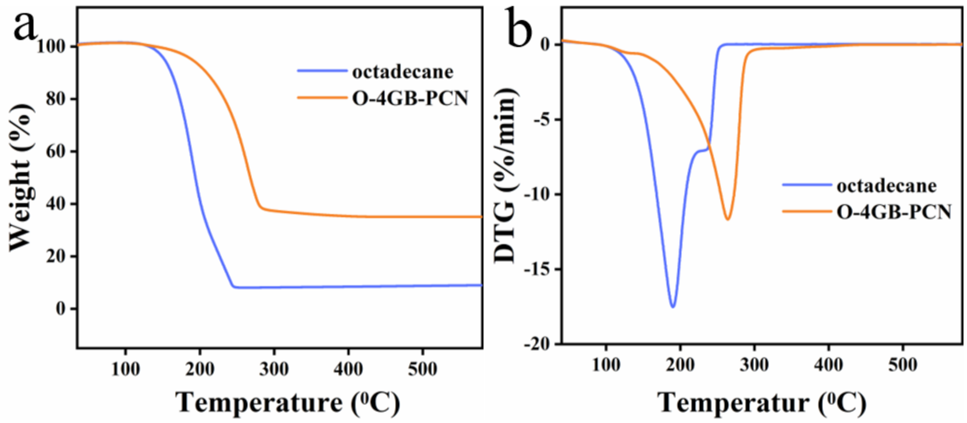

Fig. S6 (**a**) TG and (**b**) DTG curves of octadecane and O-4GB-PCN

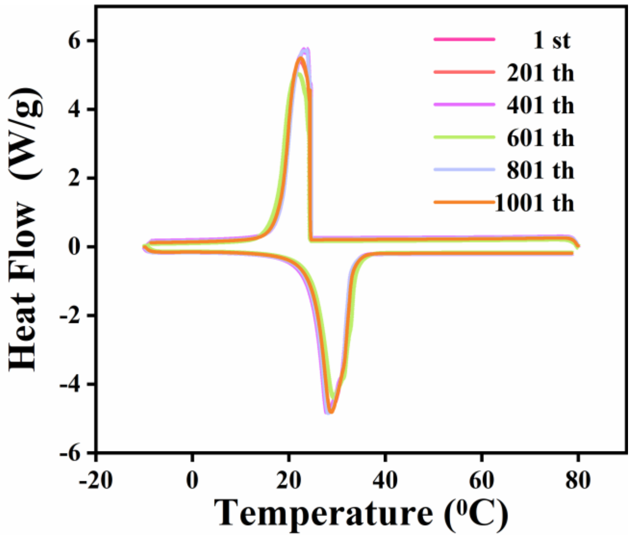

Fig. S7 Cycling stability of O-2GB-PCN

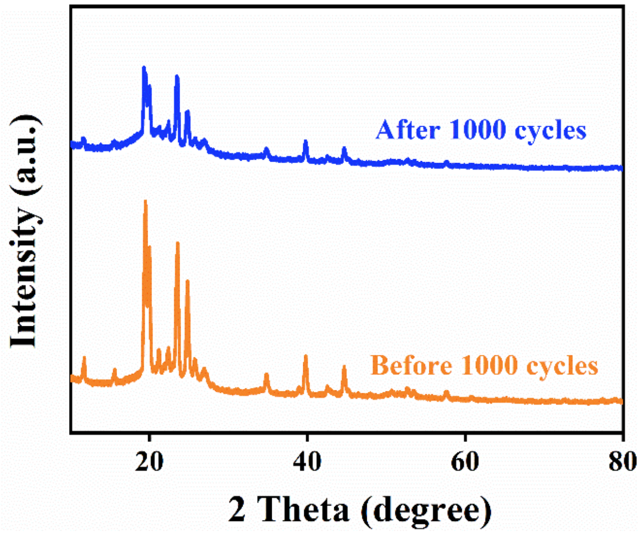

Fig. S8 XRD patterns of O-2GB-PCN before and after 1000 cycles

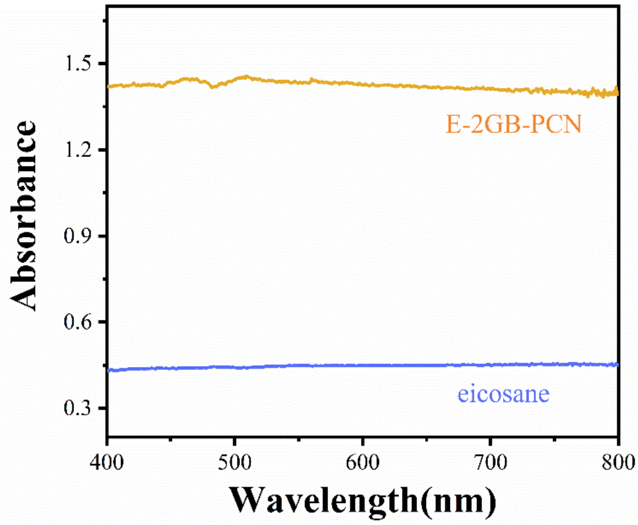

Fig. S9 UV-vis absorption spectra of eicosane and E-2GB-PCN

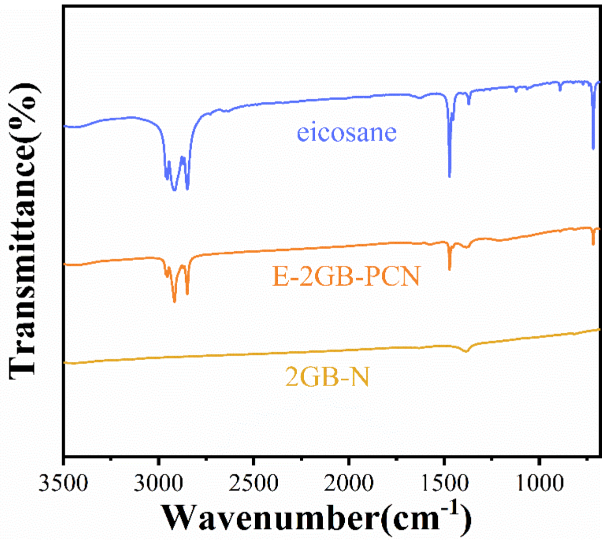

Fig. S10 FT-IR spectrum of eicosane, 2GB-N and E-2GB-PCN

Table S4 Water vapor transmission of PE, cotton and cotton-E-2GB-PCN

	$m_{(PE)}$/g	$m_{(cotton-PCN)}$/g	$m_{(cotton)}$/g
0 h	36.4125	38.4947	38.4852
8.17 h	36.4094	38.3184	38.2803
20.67 h	36.4058	38.0294	37.9663
32.08 h	36.4016	37.7634	37.6800
44.25 h	36.3977	37.5090	37.3745
56.83 h	36.3935	37.2288	37.0588

Table S5 Comparative performance of the phase change fabric in previous literatures

Sample	Diameter (μm)	Melting temperature (℃)	Enthalpy (J/g)	Loading capacity	Water vapor permeability	Refs.
PCF/MCNC	0.23	62.4<br>61.5	69.2<br>83.1	38.4%<br>46.0%	-	[S1]
PCM-30 (9 w/w% PVA)	0.75	38.46<br>37.95	84.7<br>48.7	38.3%<br>22.0%	-	[S2]
PW@H-KAF	91.8	44.4	135	72.0%	✓	[S3]
OD@F-SiO_2-PA	2.66	49.6	56.9	47.8%	-	[S4]
Polyethylene glycol/polyvinylpyrrolidone/ CNTs	4.5	51.9	77.5	35.7%	-	[S5]
Polyethylene terephthalate particles (PET)/ lauric acid (LA) /palmitic acid (PA)/	2.0	36.3	61.2	36.1%	-	[S6]
CPCF	0.15-0.4	26.1<br>25.7<br>24.6	43.4<br>56.2<br>72.3	28.1%<br>36.4%<br>46.8%	-	[S7]
Polyethylene glycol (PEG)/ polyvinylpyrrolidone (PVP)/Nano-Al_2O_3	2.66	49.6	56.9	47.8%	-	[S8]
Commercial hollow Polypropylene/PW	350	37.39	199.9	88.6%	✓	[S9]
PW@PU	2	20-35	83.96<br>120.46	41.3%<br>59.2%	-	[S10]
E-2GB-PCN	about 250	38.8	206.0	83.0%	✓	This work

Supplementary References

[S1] S. Y. H. Abdalkarim, Z. Ouyang, H.-Y. Yu, Y. Li, C. Wang et al., Magnetic cellulose nanocrystals hybrids reinforced phase change fiber composites with highly thermal energy storage efficiencies. Carbohydr Polym. **254**, 117481 (2021). https://doi.org/10.1016/j.carbpol.2020.117481

[S2] E. Zdraveva, J. Fang, B. Mijovic, T. Lin, Electrospun poly(vinyl alcohol)/phase change material fibers: Morphology, heat properties, and stability. Ind. Eng. Chem. Res. **54**(35), 8706-8712 (2015). https://doi.org/10.1021/acs.iecr.5b01822

[S3] Y. Bao, J. Lyu, Z. Liu, Y. Ding, X. Zhang, Bending stiffness-directed fabricating of kevlar aerogel-confined organic phase-change fibers. ACS Nano **15**(9), 15180-15190 (2021). https://doi.org/10.1021/acsnano.1c05693

[S4] W. Xia, X. Fei, Q. Wang, Y. Lu, M. T. Innocent et al., Nano-hybridized form-stable ester@F-SiO_2 phase change materials for melt-spun PA6 fibers engineered towards smart thermal management fabrics. Chem. Eng. J. **403**, 126369 (2021). https://doi.org/10.1016/j.cej.2020.126369

[S5] W. Zhang, X. Zhang, Y. Xu, Y. Xu, J. Qiao et al., Flexible polyethylene glycol/polyvinylpyrrolidone composite phase change fibres: Preparation, characterization, and thermal conductivity enhancement. Polymer **214**, 123258 (2021). https://doi.org/10.1016/j.polymer.2020.123258

[S6] H. Ke, Preparation of electrospun LA-PA/PET/Ag form-stable phase change composite fibers with improved thermal energy storage and retrieval rates via electrospinning and followed by uv irradiation photoreduction method. Fibers Polym **17**(8), 1198-1205 (2016). https://doi.org/10.1007/s12221-016-6456-1

[S7] W. Zhu, Y. Wang, S. Song, H. Ai, F. Qiu et al., Environmental-friendly electrospun phase change fiber with exceptional thermal energy storage performance. Sol. Energy Mater. Sol. Cells **222**, 110939 (2021). https://doi.org/10.1016/j.solmat.2020.110939

[S8] X. Zhang, B. Wu, G. Chen, Y. Xu, T. Shi et al., Preparation and characterization of flexible smart glycol/polyvinylpyrrolidone/nano Al_2O_3 phase change fibers. Energy Fuels **35**(1), 877-882 (2021). https://doi.org/10.1021/acs.energyfuels.0c03370

[S9] Q. Zhang, Z. He, X. Fang, X. Zhang, Z. Zhang, Experimental and numerical investigations on a flexible paraffin/fiber composite phase change material for thermal therapy mask. Energy Storage Mater. **6**, 36-45 (2017). https://doi.org/10.1016/j.ensm.2016.09.006

[S10] J. Wu, M. Wang, L. Dong, J. Shi, M. Ohyama et al., A trimode

thermoregulatory flexible fibrous membrane designed with hierarchical core-sheath fiber structure for wearable personal thermal management. ACS Nano **16**(8), 12801-12812 (2022). https://doi.org/10.1021/acsnano.2c04971
